# Supplementary material for: Membrane potential regulates Hedgehog signalling in the Drosophila wing imaginal disc
Source: EMBO Rep. 2021 Feb 25;22(4):e51861. doi: 10.15252/embr.202051861 (PMC8024891; doi:10.15252/embr.202051861)
Supplement: Supplementary file 1 — Expanded View Figures PDF [file EMBR-22-e51861-s001.pdf]

Expanded View Figures

Figure EV1. *rpk* knockdown decreases DiBAC staining and Rpk expression at the A-P compartment boundary.

A Expression of *rpk*-RNAi in the wing pouch decreases patterned DiBAC staining (white arrowhead).
B Expression of *rpk*-RNAi in the wing pouch decreases anti-Rpk staining anterior to the A-P compartment boundary (white arrowhead).
C, D Control discs. Increased DiBAC staining and anti-Rpk staining are visible in the centre of the wing pouch (white arrowheads).

Data information: Scale bars are 100 μm.

© 2021 The Authors

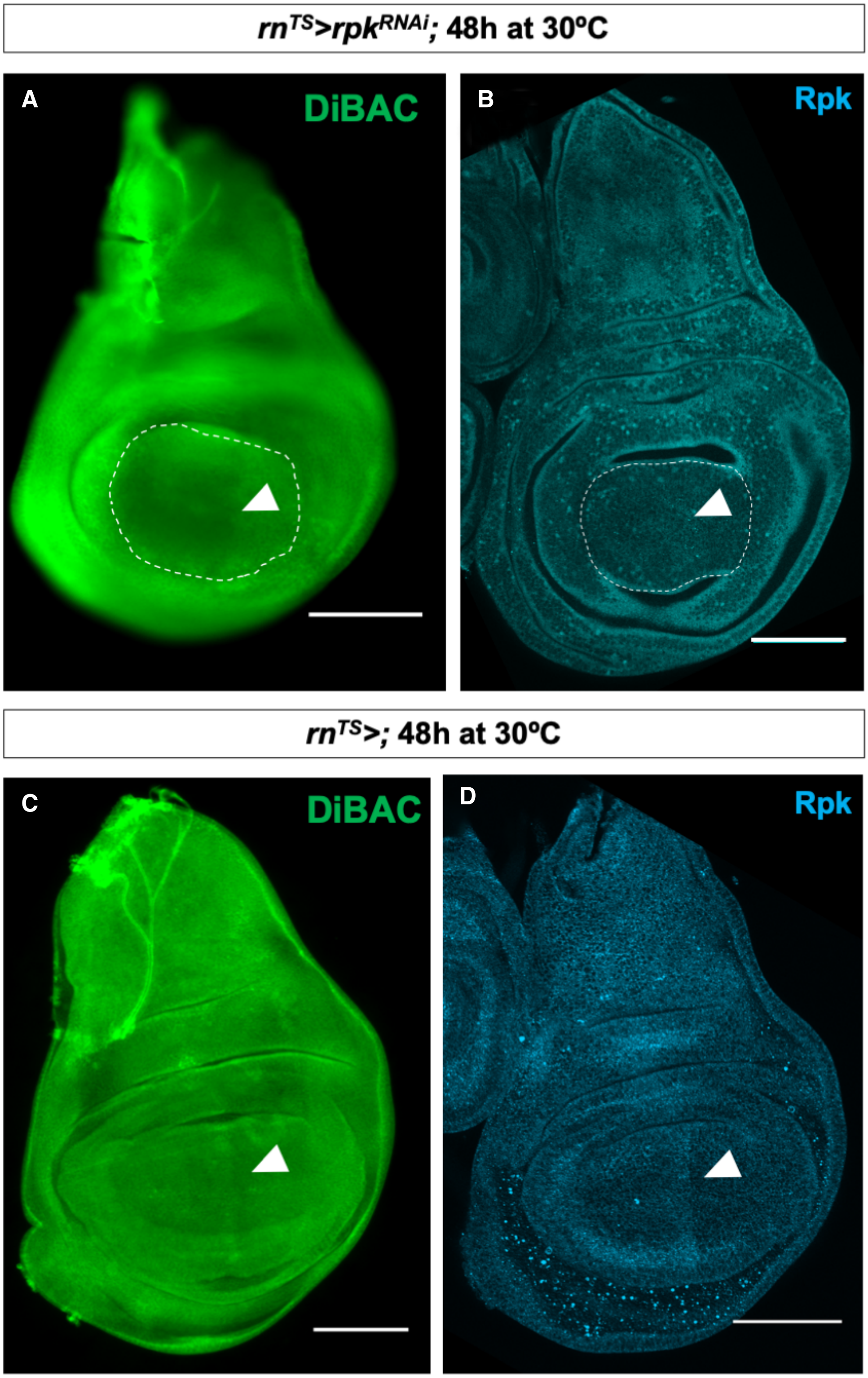
rnTS>rpkRNAi; 48h at 30ºC
A
DiBAC
B
Rpk
rnTS>; 48h at 30ºC
C
DiBAC
D
Rpk

Figure EV1.

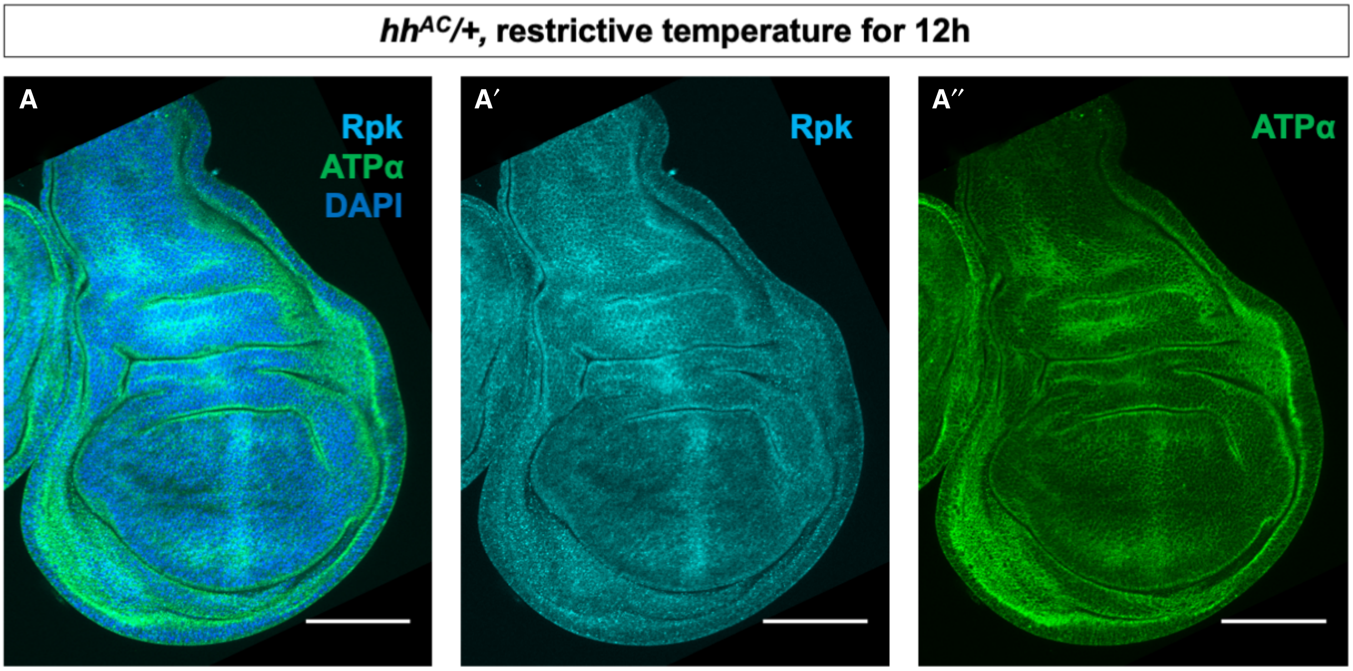

Figure EV2. Patterned Rpk and ATPα expression and membrane depolarization require Hh signalling.

A–A″ Immunostaining of discs heterozygous for the *hh*^{AC} allele and upshifted to 30°C for 12 h with antibodies to Rpk and ATPα showing elevated levels of both proteins anterior to the A-P compartment boundary. All scale bars are 100 μm.

 © 2021 The Authors

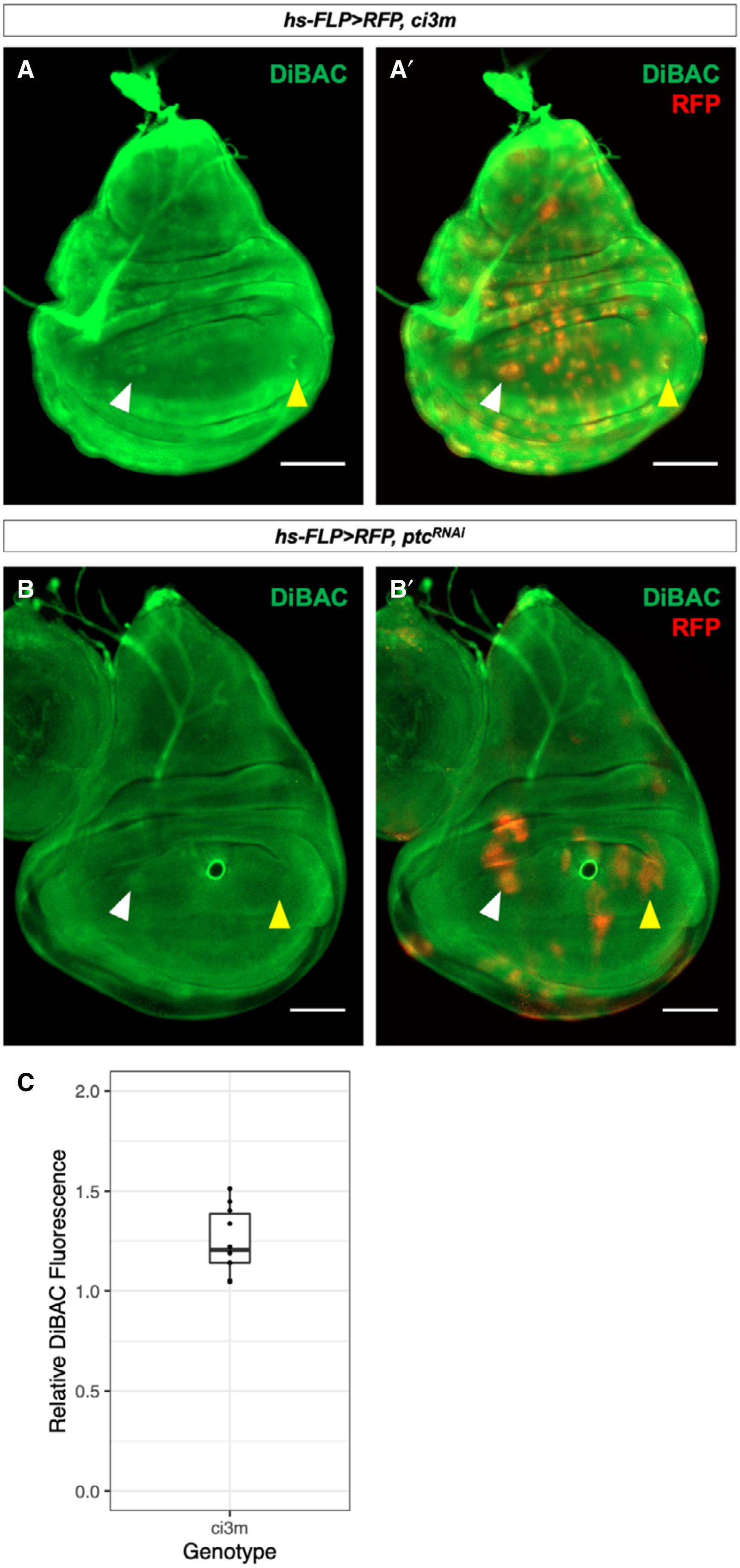

Figure EV3. Hh pathway activation results in depolarization.

A–A′ Clones of cells expressing *ci3m* accumulate more DiBAC relative to surrounding tissue in both the anterior (white arrow) and posterior (yellow arrow) compartments.

B–B′ Clones of cells expressing *ptc*-RNAi accumulate more DiBAC relative to surrounding tissue in the anterior compartment (white arrow), but not the posterior compartment (yellow arrow).

C The ratio of DiBAC fluorescence in *ci3m*-expressing clones to equally-sized regions of tissue not expressing *ci3m*. $N = 10$ clones from three imaginal discs. Individual data points are shown, as well as a box plot showing descriptive statistics. Bounds of the box show lower and upper quartiles, bar within the box shows the median of the data, and whiskers show minimum and maximum bounds of data.

Data information: All scale bars are 100 μm.

© 2021 The Authors

Figure EV4. Manipulating levels of the ion channel Rpk impacts both Hh and Wg signal transduction.

A, B Expression patterns of *ap-Gal4* (A) and *rn-Gal4* (B).

C, D Immunostaining of Smo protein (red), full-length Ci (light blue) (C–C″) and Ptc protein (D) in discs expressing $ATP\alpha^{RNAi}$ in the dorsal compartment. Brackets indicate tissue expressing RNAi and control tissue.

E–F″ Immunostaining of Smo protein (red) and full-length Ci (light blue) in (E–E″) control discs and (F–F″) discs expressing rpk^{RNAi} for 48 h before dissection.

G–H′ Immunostaining of Ptc protein in (G, G′) control discs and (H, H′) discs expressing rpk^{RNAi} for 48 h before dissection.

I–J″ Immunostaining for Wg protein in (I–I″) control discs and (J–J″) discs expressing rpk^{RNAi} for 48 h before dissection.

K–L′ Immunostaining for Cut protein in (K, K′) control discs and (L, L′), discs expressing rpk^{RNAi} for 48 h before dissection.

Data information: All scale bars are 100 μm except (D), where scale bar is 50 μm.

© 2021 The Authors

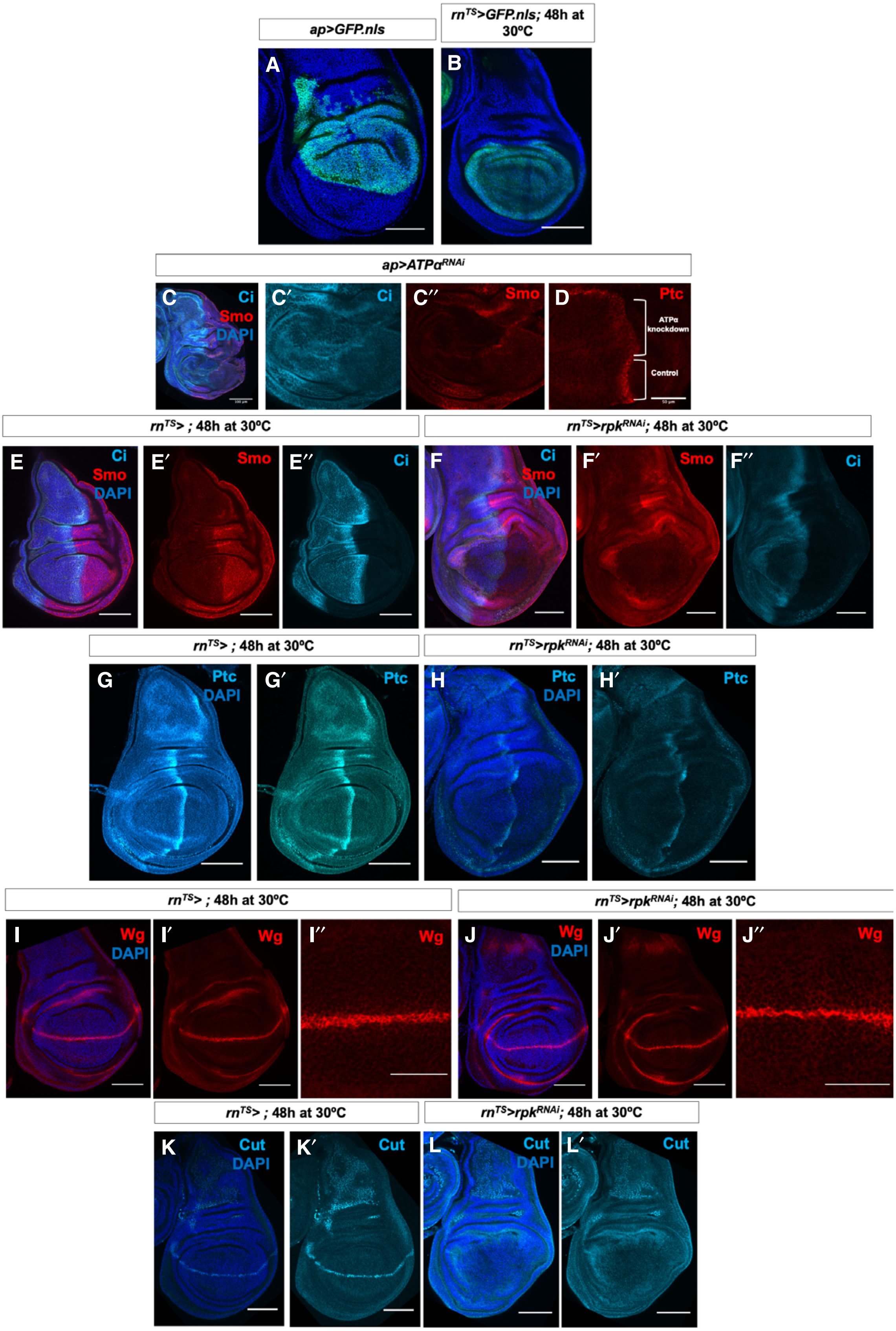

ap>GFP.nls
rnTS>GFP.nls; 48h at 30ºC
A
B
ap>ATPαRNAi
C Ci Smo DAPI
C′ Ci
C″ Smo
D Ptc
ATPα knockdown
Control
rnTS> ; 48h at 30ºC
rnTS>rpkRNAi; 48h at 30ºC
E Ci Smo DAPI
E′ Smo
E″ Ci
F Ci Smo DAPI
F′ Smo
F″ Ci
G Ptc DAPI
G′ Ptc
H Ptc DAPI
H′ Ptc
I Wg DAPI
I′ Wg
I″ Wg
J Wg DAPI
J′ Wg
J″ Wg
K Cut DAPI
K′ Cut
L Cut DAPI
L′ Cut

Figure EV4.

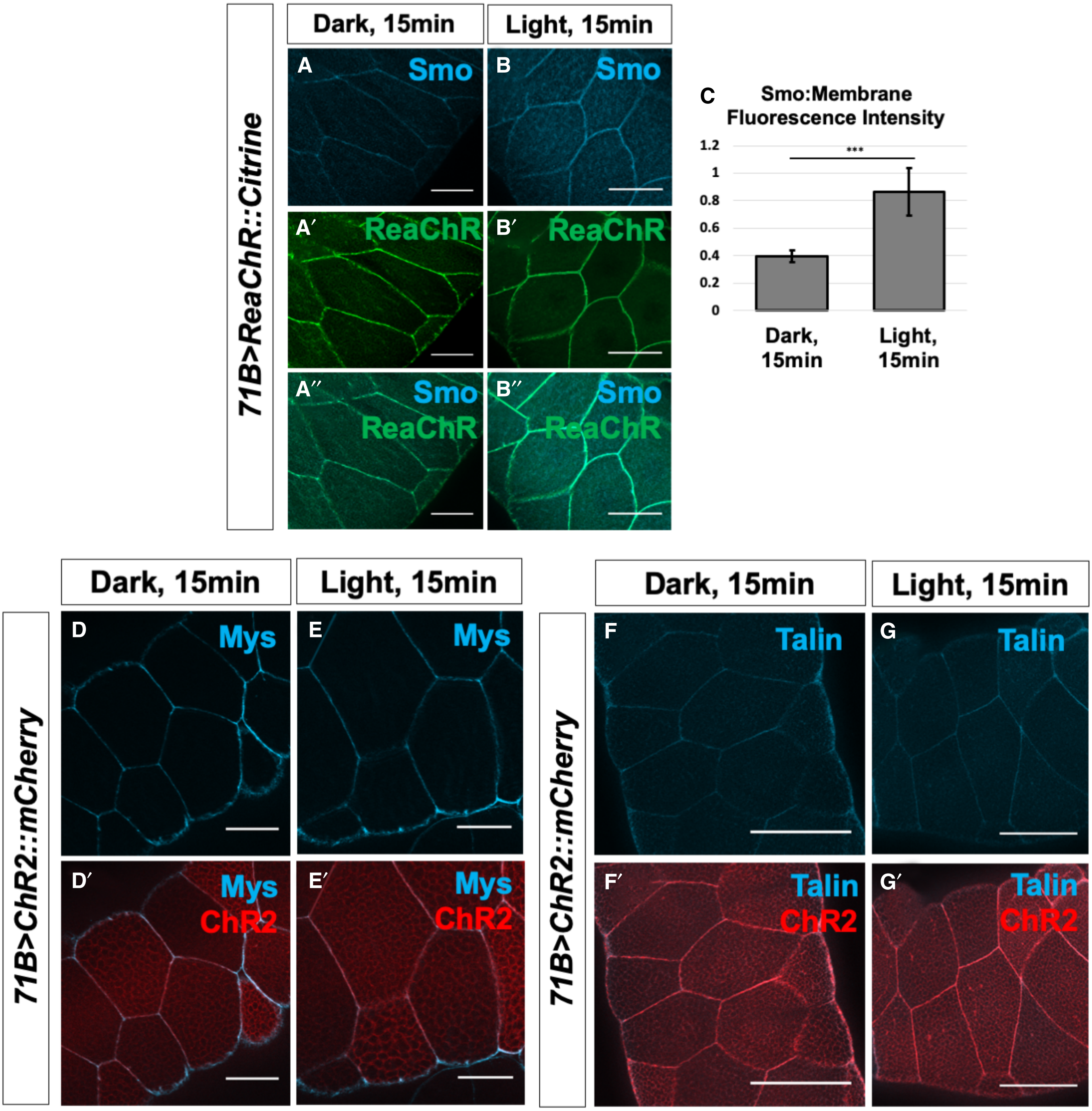

Figure EV5. Optogenetic manipulation of V_{mem} in *Drosophila* salivary glands.

A–G′ Salivary glands expressing the channelrhodopsin ReaChR were subjected to 15 min in activating light, then fixed and stained for Smo protein (A–C). $N = 9$ glands, data were compared using an unpaired *t*-test (***$P < 0.001$), error bars are standard deviations. Membrane abundance of the beta integrin subunit Mys and the integrin-associated protein Talin are not altered upon ChR2 activation (D–G′). Scale bars are 50 µm in all panels.

 © 2021 The Authors
